# Organizational responses to the COVID-19 pandemic in Victoria, Australia: A qualitative study across four healthcare settings

**DOI:** 10.3389/fpubh.2022.965664

**Published:** 2022-09-29

**Authors:** Sarah L. McGuinness, Johnson Josphin, Owen Eades, Sharon Clifford, Jane Fisher, Maggie Kirkman, Grant Russell, Carol L. Hodgson, Helen L. Kelsall, Riki Lane, Helen Skouteris, Karen L. Smith, Karin Leder

**Affiliations:** ^1^School of Public Health and Preventive Medicine, Monash University, Melbourne, VIC, Australia; ^2^Alfred Health, Melbourne, VIC, Australia; ^3^Ambulance Victoria, Melbourne, VIC, Australia; ^4^Royal Melbourne Hospital, Melbourne, VIC, Australia

**Keywords:** COVID-19, healthcare workers, mental health, occupational health, perceptions, workplace responses, pandemic

## Abstract

**Objective:**

Organizational responses that support healthcare workers (HCWs) and mitigate health risks are necessary to offset the impact of the COVID-19 pandemic. We aimed to understand how HCWs and key personnel working in healthcare settings in Melbourne, Australia perceived their employing organizations' responses to the COVID-19 pandemic.

**Method:**

In this qualitative study, conducted May-July 2021 as part of the longitudinal Coronavirus in Victorian Healthcare and Aged Care Workers (COVIC-HA) study, we purposively sampled and interviewed HCWs and key personnel from healthcare organizations across hospital, ambulance, aged care and primary care (general practice) settings. We also examined HCWs' free-text responses to a question about organizational resources and/or supports from the COVIC-HA Study's baseline survey. We thematically analyzed data using an iterative process.

**Results:**

We analyzed data from interviews with 28 HCWs and 21 key personnel and free-text responses from 365 HCWs, yielding three major themes: *navigating a changing and uncertain environment, maintaining service delivery during a pandemic*, and *meeting the safety and psychological needs of staff* . HCWs valued organizational efforts to engage openly and honesty with staff, and proactive responses such as strategies to enhance workplace safety (e.g., personal protective equipment spotters). Suggestions for improvement identified in the themes included streamlined information processes, greater involvement of HCWs in decision-making, increased investment in staff wellbeing initiatives and sustainable approaches to strengthen the healthcare workforce.

**Conclusions:**

This study provides in-depth insights into the challenges and successes of organizational responses across four healthcare settings in the uncertain environment of a pandemic. Future efforts to mitigate the impact of acute stressors on HCWs should include a strong focus on bidirectional communication, effective and realistic strategies to strengthen and sustain the healthcare workforce, and greater investment in flexible and meaningful psychological support and wellbeing initiatives for HCWs.

## Introduction

The COVID-19 pandemic has placed enormous strain on healthcare systems around the world, making great demands on healthcare workers (HCWs) and healthcare organizations (1). HCWs have been more likely to contract COVID-19 compared to the general community, and have experienced high rates of depression, anxiety, post-traumatic stress disorder (PTSD) and burnout (2–8). In response to COVID-19, healthcare organizations have needed to overhaul their standard frameworks for service delivery and workforce and infrastructure management (9, 10) to accommodate the demands of ensuring adherence to stringent infection control practices, managing an at-risk and fatigued workforce, and maintaining high quality patient care in the face of frequent worker shortages related to isolation or quarantine requirements (11–13).

With ongoing waves of infection and emerging variants of concern, it is imperative that healthcare organizations continue to improve processes and practices, and institute responsive workplace mitigation strategies designed to protect workers both physically and mentally. Existing qualitative literature has identified some pandemic-specific workplace issues that may adversely affect HCW mental health, including pandemic ill-preparedness, limited access to adequate personal protective equipment (PPE) and lack of effective communication (14–17). However, literature describing HCWs' experiences of the pandemic is generally limited to a specific healthcare setting or occupational group and few studies have explored the influence of organizational settings on self-reported pandemic experiences. We used a qualitative approach to examine individual HCW and key personnel experiences, grounded in their organizational setting. Specifically, we sought to understand how HCWs and key personnel across four healthcare settings (hospital, ambulance, aged care and primary care) perceived the approaches employed by their organizations to mitigate the impact of the pandemic on workers. We had a particular focus on the management systems, responsiveness and success of these approaches.

## Methods

This study is part of the broader COVIC-HA cohort study (8), a longitudinal, mixed methods study examining the physical, psychosocial and wellbeing impacts of the COVID-19 pandemic on a cohort of Victorian HCWs and perceptions of their organizations' responses over time. Quantitative findings from the baseline survey of this study have been described elsewhere (8). This qualitative study used thematic analysis (18) to analyse data from HCWs' free-text responses relating to a question on perceptions of organizational responses from the COVIC-HA baseline survey and data collected through semi-structured interviews with HCWs and organizational key personnel focusing on perceptions of workplace responses to the pandemic. We report our study in line with the consolidated criteria for reporting of qualitative research (COREQ) (19) and include the COREQ checklist (Supplementary Table 1).

### Setting and context

This study involved HCWs and key personnel from healthcare organizations in Melbourne, Australia. Prior to recruitment (March 2021), phased rollout of COVID-19 vaccination was underway, the first (March-April 2020) and second (June-Oct 2020) waves of COVID-19 infections in Victoria had passed, and the state was experiencing low COVID-19 case numbers and eased restrictions. During data collection (May-June 2021), there were two short lockdowns, with increased restrictions, in response to smaller COVID-19 outbreaks (Supplementary Figure 1).

### Recruitment

Recruitment for the COVIC-HA study has been described previously (8). All HCWs participating in the COVIC-HA baseline survey were given the opportunity to respond to an open-ended question relating to perceptions of their organization's response to the COVID-19 pandemic.

We recruited HCW interview participants from the COVIC-HA Study cohort, with the aim of capturing a diverse set of experiences from across the healthcare sector. Participant consent to be contacted for interviews was obtained during completion of the COVIC-HA baseline survey. HCW interview participants were recruited prior to key personnel. Informed by demographic data collected at enrolment, we used purposive sampling to ensure diversity across organizations and healthcare settings. We used a maximum variation sample approach to select participants of different ages, genders, professions, healthcare settings and levels of COVID-19 exposure (e.g., infection, furlough).

We recruited key personnel interview participants from those nominated by CEOs, practice managers or site principal investigators at participating organizations. We sought to recruit those with an in-depth knowledge of their organization's pandemic preparedness and response plans. At each participating organization, we aimed to select at least one person in a senior clinical role (e.g., medical executive, area manager or practice manager) and one in a senior occupational health and safety or infection control role. Some key personnel interview participants (e.g., general practice owners) had roles as both HCWs and managers and some HCW interview participants also held management roles.

Two research staff (JJ & AM) phoned hospital, ambulance and aged care participants, following up by email for non-response. Primary care participants were contacted by the primary care project manager (SC). Participants who expressed interest received an explanatory statement and were invited to an interview. Guided by the concept of information power (20), we aimed to recruit a diverse sample of participants across participating healthcare organizations of a size sufficient to provide robust information directly related to our research question. Braun and Clarke consider the concept of information power–which indicates that the more relevant information a sample holds, the fewer participants are needed–to offer a useful alternative to data saturation in informing sample size considerations in reflexive thematic analysis (21). Given that our study had a relatively narrow aim, that participants shared specific experiences of working in healthcare during the COVID-19 pandemic, that the dialogue quality in interviews was expected to be strong, and that free-text response data from a large group of HCWs had already been collected, we concluded that a sample size of at least 24 HCWs and 20 key personnel would likely hold appropriate information power for analysis. At least 3 HCWs and 2 key personnel were recruited from each hospital, aged care and ambulance organization; primary care participants were selected from across 11 practices.

### Interview guides

We developed and piloted separate semi-structured interview guides for HCWs and key personnel based on expert knowledge and prior evidence (22, 23). Planned topics included challenges faced during the pandemic, perceptions of organizational responses, and positive experiences (Supplementary Files 1, 2). Key embedded questions were “How did management respond?” and “How successful were responses in mitigating the challenges?” Interview guides were flexible, enabling interviewers to rephrase questions or alter their sequence depending on individual interview circumstances.

### Data collection

All free-text responses to a question in the COVIC-HA baseline survey (“What resources or supports would you like to see your organization put in place to support you during the COVID-19 pandemic or any future crisis events?”) were extracted, de-identified, and included in analysis.

Interview data were collected by six researchers (five female, one male, all experienced in qualitative interviewing) *via* an online video platform (Zoom Video Communications, Inc) or telephone between 3 May and 22 July 2021 (24). Verbal consent was obtained before each interview. Interviews were audio-recorded with participants' permission. HCW interview participants (not key personnel) were offered a digital $50 gift card in recognition of their time and any inconvenience. Audio files were transcribed by a professional transcription service, reviewed by a member of the research team, and de-identified before analysis.

### Data analysis

**Figure 1:**
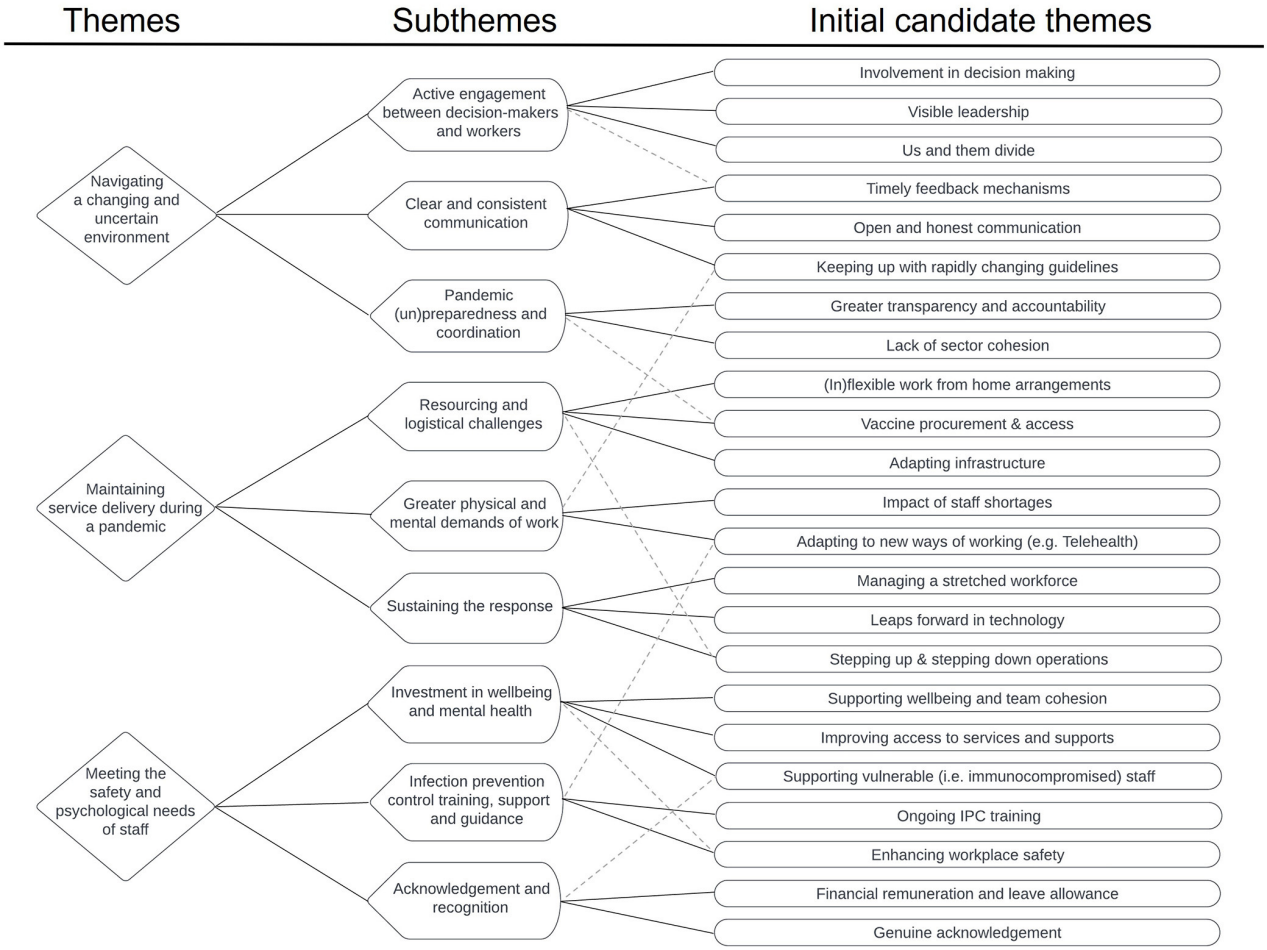
Thematic tree of findings showing the evolutionary process of theme conceptualization. *Dotted lines indicate examples where an initial candidate theme was related to multiple subthemes.

Transcripts and free-text responses were examined using a reflexive and primarily inductive approach to thematic analysis, following the framework outlined by Braun and Clarke (18). NVivo (v12) (25) was used for data management. In line with reflexive thematic analysis (26), research questions were addressed within a paradigmatic framework of interpretivism and constructivism, with data collected and analyzed in a manner that respected and expressed the subjectivity of participant's accounts, while acknowledging and embracing the reflexive influence of researchers' interpretations. An experiential orientation to data interpretation was adopted in order to emphasize the true meaning of responses as ascribed by participants. One primary interviewer (SM, PhD public health) undertook the initial analysis following Braun and Clarke's (18) six phase approach to thematic analysis. Data familiarization involved actively listening to each interview recording and reading through all interview transcripts and free-text responses multiple times whilst making preliminary notes. Data from each source (HCW interviews, key personnel interviews and free-text survey responses) was then analyzed separately using a flexible and organic coding approach that supported the active creation of themes. Initial candidate themes from each dataset were then collated and clustered into broader patterns and themes. Mind maps and tables were used to explore and examine relationships and levels of themes within each of the datasets. The collated themes were then discussed with the wider research team (including JF, PhD, psychology; MK, PhD, psychology; HK, PhD, public health and KL, PhD, medicine) to sense-check ideas and achieve richer interpretations of meaning. A collaborative and iterative approach was then used to conceptualize the final theme and sub-theme structure (Figure 1).

### Ethics

This study was approved through the Victorian Streamlined Ethical Review Process (SERP: Project Number 68086) and registered with ANZCTR (ACTRN12621000533897).

## Results

### Survey free-text participants

Of the 989 HCWs who completed the COVIC-HA baseline survey, 365 (37%) provided free text responses (WFT) describing the resources or supports they would like their organization to adopt. Characteristics of those providing free text responses were similar to all survey participants; most worked in a hospital (81%) or ambulance (16%) setting and the majority (75%) were female (Table 1).

**Table 1:** Participant characteristics.

			**Free text respondents**	**Interview participants**	
**Characteristic**	**Detail**		**HCWs** **(*n* = 365), *n* (%)**	**HCWs** **(*n* = 28), *n* (%)**	**Key Personnel** **(*n* = 21), *n* (%)**
Profession^#^	Nurse		131 (36)	6 (21)	-
	Doctor		39 (11)	5 (18)	-
	Allied health		65 (18)	5 (18)	-
	Paramedic		51 (14)	3 (11)	-
	Other*		79 (22)	9 (32)	-
Gender^∧^	Female		273 (75)	19 (68)	12 (57)
	Male		90 (24)	9 (32)	9 (43)
	Non-binary		2 (1)	-	-
Healthcare setting	Hospital		294 (81)	14 (50)	8 (38)
	Ambulance		60 (16)	3 (11)	2 (10)
	Primary care		8 (2)	7 (25)	5 (24)
	Aged care		3 (1)	4 (14)	6 (28)
Age group	20–29		44 (12)	2 (7)	-
	30–39		89 (24)	5 (18)	-
	40–49		91 (25)	9 (32)	-
	50–59		89 (24)	8 (29)	-
	60–69		47 (13)	4 (14)	-
	70–79		4 (1)	-	-
	80+		1 (1)	-	-

^#^Key personnel included medical executives, area managers, practice managers and those in a senior occupational health and safety or infection control role. ^*^Includes personal care attendants, clinical and laboratory technicians, clerical and administrative staff, and other support staff. ^∧^No interviewparticipants declared they were non-binary, gender diverse, or other in survey responses.

### Interview participants

We conducted interviews (lasting between 18 and 60 min) with 28 HCWs (HCWI) and 21 key personnel (KPI). Table 1 shows the characteristics of participants.

### Key themes and sub-themes

We identified three major themes and nine sub-themes from the data (Figure 1). Themes are presented in italics and sub-themes are underlined in the text. *Navigating a changing and uncertain environment* consistently emerged as an important theme for HCWs and key personnel as they learned to adapt to the dynamic and uncertain environment of the pandemic. The challenges inherent in *maintaining service delivery during a pandemic* were a source of much discussion. *Meeting the safety and psychological needs of staff* was seen as a particularly priority by both HCWs and key personnel. Key elements of organizational responses that were perceived favorably by HCWs and suggestions for future responses to COVID-19 and other crisis events are summarized in Table 2.

**Table 2:** Summary of organizational innovations and suggestions for the future.

**Theme**	**Organizational innovations**	**Suggestions for the future**
Navigating a changing and uncertain environment	Expert-led sessions to address sources of staff anxiety (e.g., vaccine safety concerns)	Streamlined information processes and centralized sources of information
	Visible leadership (e.g., on-site visits, video messages, presence at forums) Open forums where HCWs can raise concerns and ask questions	Greater consultation with and involvement of HCWs in decision-making (at organizational and government levels) Hazard pay and increased leave entitlements during crisis events
Maintaining service delivery during a pandemic	Innovations in the utilization of technology in the workplace (e.g., telehealth)	Strengthening the workforce (e.g., increasing staff numbers, improving staff ratios)
	Management of vulnerable staff through redeployment or support to work-from-home	Greater work flexibility including support for work-from-home arrangements where feasible
Meeting the safety and psychological needs of staff	Strategies to reinforce and increase confidence in infection control practices (e.g., refresher training, PPE safety spotters) Activities aimed at lifting morale and keeping people connected (e.g., team-building exercises, song of the day)	Practical forms of acknowledgment (e.g., free meals while on duty) Greater investment in and access to staff wellbeing initiatives, such as wellbeing officers, wellness checks and debriefing opportunities

### Navigating a changing and uncertain environment

In a rapidly changing and uncertain pandemic environment, HCWs valued leaders who were visible and accessible (e.g., *via* on-site walkthroughs, staff forums or video updates) and viewed active engagement between decision-makers and workers as highly important. While HCWs appreciated opportunities to voice concerns *via* platforms such as regular open forums and live Q&A, it was important to them that their concerns were addressed. Having a platform to voice concerns was viewed as pointless if organizations did not act in response.

*If they just listened early on to what the problems were… they might have been able to address them and keep morale going. (HCWI, paramedic, ambulance, male, aged 50–59 years)*

Many HCWs felt that more direct consultation with, and increased HCW involvement in, decision-making was needed. Insufficient engagement with HCWs undermined trust and was perceived to lead to impractical policies. Combined with a sense that leaders were not necessarily facing the same risks as workers, this contributed to an us-and-them dynamic between HCWs and management.

*A lot of decisions were being made by executives that were sitting at home and didn't have an idea of what things actually looked like on the ground. (HCWI, doctor, hospital, male, aged 40–49 years)*

Key personnel reported that efforts made by leaders to be visible, accessible and respond to staff concerns were appreciated by HCWs and “built trust” (KPI, hospital, female) between staff and management.

*I think they appreciate that we're not just directing from afar, but we're going there and talking to them, and saying, I see that doesn't work here so let's change it. (KPI, primary care, female)*

HCWs and key personnel viewed clear and consistent communication as vital to pandemic management, with important aspects including timeliness, accuracy, and transparency, particularly in the face of uncertainty. HCWs' perceptions of how well their organizations kept them informed were mixed, ranging from “absolutely brilliant” (HCWI, nurse, hospital, female, aged 40–49 years) to “a constant stream of information that was hard to keep up with” (HCWI, paramedic, ambulance, male, aged 50–59 years). HCWs also spoke of difficulties working with inconsistent policies across multiple healthcare services.

*[It] was very frustrating because, like I said, it didn't feel like we were all working for the same health system; everyone had slightly different ideas. (HCWI, paramedic, ambulance, male, aged 50–59 years)*

HCW suggested that information sharing could be improved through the provision of regular updates that were clear, concise, and available “in one place on one platform” (WFT, allied health, hospital, female, aged 30–39 years).

Several key personnel claimed that “staff quickly got oversaturated with incoming information” (KPI, hospital, female) and found it challenging to ensure that HCWs had the most current information without overwhelming them. Strategies employed to streamline messaging included weekly updates and information distribution by managers who could filter it appropriately to relevant departments. Key personnel from all four organizational settings stated that communication challenges were amplified by rapidly changing policies and directives from government health agencies, and slow responses to requests for clarification. Communication systems and channels with health agencies were seen as fragmented, and it was not always clear who was responsible for making decisions.

*It was quite difficult to keep up with what was going on, and make sure that everybody was operating using the most current information. (KPI, aged care, female)*

Key personnel from all settings saw value in within-sector collaboration and learning from the experiences of other organizations. Examples included Victorian hospital intensive care directors establishing regular meetings to discuss patient flow and COVID-19 management (KPI, hospital, male & KPI, hospital, male), and general practitioners sharing experiences and policies from other practices (KPI, primary care, female). There was a sense that greater collaboration between organizations and government was needed, particularly in the early phase of the response and in aged care.

*I think the aged care sector was almost caught sleeping. … If there had been better proactiveness, better collaboration from the sector, and from state and Commonwealth departments, we probably would have managed COVID a lot better in the early stages. (KPI, aged care, male)*

Perceived lack of transparency regarding HCW COVID-19 infections was mentioned by several HCWs. One infected HCW indicated a need for improved reporting and investigation of work-related COVID-19 infections:

*I was exceedingly disappointed and frustrated at the lack of review of what happened. … There was no effort to learn. (HCWI, nurse, hospital, female, aged 40–49 years)*

Key personnel noted that efforts to manage and investigate HCW exposures were initially hampered by delays at the Department of Health, leading several organizations to initiate their own processes.

*We had many instances where [close contact] staff members were only getting their initial contact from the Department of Health contact tracing at day 10. … We made a decision to do our own contact tracing and take our own steps to furlough staff. (KPI, ambulance, male)*

Key personnel also called for better digital tools to integrate contact tracing and outbreak investigation systems across organizations and with the Department of Health.

Pandemic (un)preparedness was a major challenge for organizations, representing a particularly prominent sub-theme arising from key personnel interviews. Across all settings, key personnel identified that existing pandemic plans and policies were not fit for purpose, leaving their organizations underprepared for the pandemic.

*There were policies written, there was some training in frontline areas, but not widespread training or a mock disaster plan. (KPI, hospital, male)*

There was also a strong sense from key personnel that government health agencies were unprepared for and inappropriately equipped to respond to a pandemic. Perceived limitations included a lack of health expertise among decision-makers, inadequate consultation with healthcare organizations and HCWs on the frontline, “mixed messaging” (KPI, aged care, male) and directives that were “impossible, impractical or frankly, incorrect” (KPI, ambulance, male).

Some HCWs also expressed concerns regarding organizational and governmental under preparedness, and felt that not enough was learnt from international experience:

*I thought that it was lucky for Australia to have that kind of lag time.... I was very upset when things could have been done, which weren't. … it's like gee, guys, you don't have to reinvent the wheel. (HCWI, doctor, primary care, female, aged 50–59 years)*

Many HCWs and key personnel perceived the COVID-19 vaccine rollout in Victoria to be slow, disorganized and poorly communicated. While hospital HCWs were generally positive about the vaccine rollout, HCWs from other settings (ambulance, aged care and primary care) reported challenges in vaccine access, particularly early on. Exclusion of some HCW groups from the initial rollout phase and the onus placed on workers to source vaccines outside work were raised as key issues. Ambulance key personnel reported being “*forgotten in that first round … and when we were included … vaccination centres were turning our people away*” (KPI, ambulance, male). Key personnel from private hospitals that were unable to access mRNA vaccines felt that their staff were “*treated as second-class healthcare workers”* (KPI, hospital, male). Aged care key personnel expressed frustration that the government did not include aged care staff in the vaccine rollout to aged care residents; this led one organization organization to “*engage a third-party provider to roll out all our vaccinations for staff* ” (KPI, aged care, male).

Key personnel across hospital, ambulance and aged care settings also noted that pre-existing governance and command structures were ill-suited to the rapid decision-making requirements of the dynamic pandemic environment and needed to be adapted to enable decisions to be made and implemented quickly.

*We learnt a lot from the structure we did set up because it was as effective as we've ever seen in terms of getting stuff done. (KPI, ambulance, male)*

### Maintaining service delivery during a pandemic

HCWs and key personnel frequently described challenges in maintaining service delivery and quality patient care in the face of resourcing and logistical challenges that arose during the pandemic. However, some also saw the pandemic as an opportunity to promote the uptake and expansion of digital technologies such as Telehealth.

Key personnel were grateful when organizations opted to overcome standard governance and budgetary constraints to enable rapid infrastructure changes, facilitate efficient resource mobilization or meet additional needs. Such measures included increasing bed capacity with a “*pop-up ICU”* (KPI, hospital, male), “*hiring contract cleaners”* (KPI, ambulance, male) to meet additional cleaning demands and “*upgrades to software and communication systems”* (KPI, aged care, female).

From a HCW perspective, a benefit of the pandemic was that it “*literally got us up and moving in relation to technology”* (HCWI, other role, hospital, female, aged 50–59 years), with widespread uptake of telehealth and innovations such as electronic prescribing, virtual meetings and video chats.

*One of the best things that they ever did in aged care was to bring in video chats. … I saw one man who hadn't spoken to his children in 3 weeks.... Then he had the video, and he saw his grandchildren, and his face lit up. (HCWI, other role, aged care, male, aged 40–49 years)*

Key personnel from all settings, particularly the private sector, said that COVID-19 had adversely affected the business of their organization, given costs incurred and disruptions to usual services including “*mandated reductions in elective surgery”* (KPI, hospital, male). Key personnel also cited differential treatment of public and private health services, such as poorer access to personal protective equipment (PPE) and vaccine stockpiles in the private sector. In primary care, key personnel indicated that their practices faced a “*dramatic drop in revenue”* (KPI, primary care, female) as a result of billing rules for remote consultations, a decrease in preventive and routine care visits, and the costs of setting up telehealth capacity. Primary care key personnel also spoke of logistical challenges and insufficient compensation for setting up practices as vaccination centers.

Both key personnel and HCWs commented on greater physical and mental demands of work and reduced efficiency in the pandemic environment. Contributing factors included additional “*administrative demands”* (KPI, primary care, male), additional infection control protocols (e.g., donning and doffing PPE, additional cleaning) and the increased “*emotional load”* (HCWI, nurse, hospital, female, aged 40–49 years) of providing care to patients and residents separated from family and friends.

*I've had debriefs with colleagues that have been changed by the experience, who were nursing COVID patients whilst their loved one was outside the window, in the pouring rain, balling that they couldn't visit them even though they were dying... You're not going to get over that. (HCWI, doctor, hospital, male, aged 40–49 years)*

Key personnel across all settings reported that these challenges were intensified by difficulties finding sufficient staff to meet demands. This was compounded by directives to limit staff movement across sites, frequent episodes of HCW furlough/isolation, and loss of the medical and allied health student workforce. Key personnel noted that it was often necessary to hire additional agency staff and surge workforce, and to redeploy staff from other areas or restructure roles for vulnerable staff, “*creating new jobs for them … or redesign the jobs they had”* (KPI, aged care, male). Incentives (e.g., hotel accommodation, transport) were offered to interstate workers from areas with lower case numbers to assist. Inadequate staffing was particularly challenging for the aged care sector with a work environment “*already understaffed … and massively depleted in the early stages”* (KPI, aged care, male).

Sustaining the response emerged as an important sub-theme among responses provided by both HCW and key personnel interviews. Several key personnel observed that HCWs' initial response to the pandemic had been positive, with staff “*keen to help … [and] rising to the occasion”* (KPI, hospital, female). However, over time, key personnel across all settings felt that people were tiring and losing energy and commitment. Key personnel described the challenges inherent in “*sustaining an emergency response”* (KPI, ambulance, male) and transitioning back to business as usual, while still maintaining the capacity to go “*from full activity to low activity”* (KPI, hospital, male) as needed. Key personnel reflected on the increasing challenge of maintaining and motivating a stretched and fatigued workforce.

*As outbreaks have progressed … complacency creeps in. Staff become tired. Staff become sick of having to put on PPE. (KPI, aged care, female)*

HCWs said that the mental health impacts of the pandemic had shifted as the pandemic progressed. Initially, there was a prevailing sense of “*anxiety and fear”* (HCWI, doctor, primary care, female, aged 30–39 years) of what might come. As the pandemic progressed, fatigue and burnout became more prevalent.

*I'm normally a very caring person but I feel I no longer care and have minimal job satisfaction due to burnout and fatigue. (WFT, paramedic, ambulance, male, aged 30–39 years)*

### Meeting the safety and psychological needs of staff

HCWs discussed the need for greater investment in wellbeing and mental health, citing the challenges of working in a pandemic setting, including the increased physical and emotional demands, the fear of passing COVID-19 on to their families, and the anguish of being separated from family and friends who were interstate and overseas. In this environment, HCWs had increased need for team relationships and interpersonal support. While several HCWs approved of organizational efforts to boost morale, such as virtual trivia and an initiative that asked staff to “*submit their ‘song of the day”* (HCWI, nurse, hospital, female, aged 40–49 years) to lift spirits, others reported increasing professional isolation as restrictions on gatherings, density limits, and COVID-19 transmission fears limited opportunities to interact with colleagues in work and social settings.

*We're a very collegiate clinic usually. We have lots of meetings and we're always knocking on each other's doors, but that kind of stopped during COVID, trying to keep everyone separate in this fear of if one person gets infected, they'll infect everyone. (HCWI, doctor, primary care, female, aged 30–39 years)*

Key personnel in all settings acknowledged the mental health burden faced by HCWs working through the pandemic and expressed concern for staff welfare. Staff forums, COVID-19 support lines, and Employee Assistance Programs (EAP) were commonly cited as strategies employed by organizations to support the mental health and wellbeing of staff.

HCWs' opinion of EAPs were mixed. While some found them helpful and supportive, there was a strong sentiment (especially in free-text responses) that existing services were insufficient. Examples cited included the limited number of counseling sessions (typically 4–6), lack of on-site or after-hours access, gaps in eligibility for access (e.g., for contractors and casuals), external providers being insufficiently familiar with onsite working conditions, and services being unable to keep up with demand. HCWs said that organizations needed to “*be proactive in regard to employee mental health”* (WFT, paramedic, ambulance, male, aged 30–39 years), and make “*an investment into the psychological wellbeing of staff now and into the future”* (HCWI, doctor, hospital, male, aged 40–49 years). Suggestions for improvement included better access to EAP via increased sessions and extended hours; introduction of staff wellbeing officers, “*wellness checks”* (WFT, other worker, aged care, male, aged 40–49 years), and debriefing/counseling training for key management staff. HCWs also cited a need for improved break facilities, given long hours spent in PPE and density limits on existing spaces.

Both HCWs and key personnel considered infection prevention and control training, support and guidance to be key aspects of organizational responses to the pandemic. Access and safety concerns about PPE were frequently raised by key personnel and HCWs and in free text. Initial HCW concerns about PPE shortages and perceived poor quality appeared to be largely addressed as the pandemic progressed and as fit-testing was introduced. However, constant policy changes and differences in the level and type of PPE available to different worker groups and at different organizations undermined workers' confidence in the appropriateness of the PPE available to them, causing them to question whether they were in the “right” PPE. Key personnel identified PPE-related “*confusion and anxiety”* among HCWs (KPI, hospital, male) as a major challenge in the early stages of the pandemic, and that it was exacerbated by mixed messages from government:

*We were trying to follow the bouncing ball of the Department [of Health], but it was impossible.... If you picked up the guidelines one night and then you looked the next night … it'd be different again. (KPI, ambulance, male)*

Key personnel reported an early realization that many staff lacked infection control knowledge and skills, suggesting that existing training was insufficient.

*Before COVID, we assumed that PPE was a simple thing, that staff were using it all the time, … but what we discovered is that they really didn't have the skills. (KPI, hospital, female)*

This necessitated rapid development and rollout of appropriate training and resources. Key personnel also described implementing strategies to monitor and support uptake of, and adherence to, PPE training. These included video monitoring of high-risk environments in hospitals and the inclusion of dedicated PPE monitors (spotters) to “*ensure that staff are … using the appropriate PPE and donning and doffing correctly”* (KPI, hospital, female). One key personnel described this strategy as “*probably the single best thing we've done”* (KPI, ambulance, male). Hospital key personnel reported that the implementation of government-mandated fit-testing programs added another layer of protection and reassurance to HCWs, despite challenges in equipment procurement and appointment scheduling. However, several key personnel noted the absence of supports for PPE training and fit testing in primary care.

HCWs appreciated organizational efforts to increase the standard of infection control in the workplace, including PPE monitors and clinical support phone lines. Despite frustration with the need for “*constant donning and doffing”* and the discomfort of working in PPE, staff recognized the need for such policies and felt “*safe to do the work”* (HCWI, other worker, hospital, female, aged 50–59 years) because of increased monitoring of infection control practices and “*constant training and retraining”* (HCWI, aged care, male, aged 40–49).

While key personnel indicated that some HCWs were eager to get vaccinated and conveyed “*psychological relief at feeling protected”* (KPI, hospital, male), many also described a need to address vaccine misinformation and HCW safety concerns. This was particularly true for aged care following the implementation of vaccine mandates:

*There is feedback coming through that staff are being forced now to get a vaccination. If they don't get a vaccination, they can potentially lose their job…. There is a little bit of angst in that space. (KPI, aged care, male)*

Shifting eligibility criteria and negative media reports undermined vaccine confidence amongst some HCWs, leading them to delay vaccination, and concerns were raised about vaccine side-effects, potential impacts on pregnancy and fertility, and the thoroughness of vaccine testing. Hospital key personnel discussed the benefits of expert-led forums to combat misinformation, address vaccine safety concerns, and communicate to staff that “*the vaccine is safe and necessary”* (KPI, hospital, male).

HCWs appreciated acknowledgment and recognition of their hard work and sacrifices. They valued genuine expressions of acknowledgment from senior leadership and practical expressions such as the provision of meals during long shifts.

*Even though it was hard work, it was nice to be appreciated, (HCWI, nurse, primary care, female, aged 30–39 years)*.

However, expressions of acknowledgment that were not accompanied by efforts to improve working conditions or increase support were seen as disingenuous:

*It's just all, clap the healthcare workers, and then you get on with the job. (HCWI, doctor, hospital, male, aged 40–49 years)*

HCWs described unsustainable workloads and feelings of burnout from operating within a stretched workforce. Additional burdens on those with caring responsibilities, such as home-schooling, were also noted. There were calls for increased “*financial support”* (WFT, doctor, primary care, female, aged 50–59 years), “*more sick leave and carers leave”* (WFT, paramedic, ambulance, female, aged 30–39 years) to cover COVID-19 related absences, particularly for contractors and casual workers; and “*hazard pay”* (WFT, nurse, hospital, male, aged 30–39 years) in recognition of challenging working conditions (e.g., risk of COVID-19 transmission, long hours in PPE, inadequate staffing). There were also calls for “*more flexible rostering”* (WFT, paramedic, ambulance, female, aged 40–49 years), “*more staff”* (WFT, allied health, hospital, female, aged 40–49 years), “*better staffing ratios”* (WFT, nurse, hospital, female, aged 40–49 years), and greater “*workplace flexibility”* (HCWI, doctor, hospital, male, aged 40–49 years) including the option to work from home for telehealth consultations or “*non-clinical components of work”* (WFT, allied health, hospital, female, aged 30–39 years).

## Discussion

The COVID-19 pandemic has challenged HCWs and healthcare organizations in Australia and internationally (2, 27). Our study provides in-depth insights into the challenges and successes of organizational and workplace responses in the uncertain environment of a global pandemic by drawing upon the organizational experiences and perceptions of HCWs and key personnel across four healthcare settings in Melbourne, Australia. We identified three major themes from participant responses, primarily centered around navigating a changing and uncertain environment, maintaining service delivery during a pandemic, and meeting the safety and psychological needs of staff. We identified organizational responses that HCWs valued and felt contributed to a better working environment. These were typically proactive initiatives and recognized the contributions and concerns of staff in a meaningful way. We also identified some similarities and differences in key personnel and HCW experiences across the four healthcare settings.

In the face of uncertainty and rapidly evolving evidence and policies, HCWs identified reciprocal communication between management and workers as important elements of organizational responses to support workers and alleviate pandemic-induced pressures. Timely and honest communication were viewed as central to effective management. However, HCWs were often overwhelmed by too much information and expressed a desire for more streamlined information-sharing and centralized information sources. Frequent changes in policy directives and differences across services, particularly in relation to PPE, led HCWs to question whether they were safe at work, mirroring findings from other recent studies (28). Keeping HCWs informed and up to date can help to alleviate anxiety and provide a sense of agency, which is of particular importance in an emergency pandemic scenario (29). Consistent with previous studies, HCWs in our cohort felt supported and valued by managers who they perceived to be visible, approachable and in touch with what was happening “on the ground” (30, 31). Recent findings suggest that leaders' physical presence at the workplace increases trust, and signals solidarity and risk-sharing in a stressful and hazardous working environment, and empathetic leadership eases staff anxiety (31, 32). In contrast, staff often feel devalued when there is misalignment between the reality of those working ‘on the ground' and the efforts of managers who are not seen to be doing the same (17). Future workplace policy decisions in healthcare should aim to include a strong focus on bidirectional communication between workers and managers, and greater HCW consultation and involvement in decision-making to facilitate a healthy and responsive workplace culture and ensure preparedness for future public health emergencies.

Our findings, along with those of others (33), suggest that the pandemic was a catalyst to accelerating HCWs' and healthcare organizations' uptake of digital technologies (such as electronic prescribing, telehealth, and virtual meetings) by overcoming barriers that typically impede change processes. However, gaps in IT systems were also described, such as the lack of integrated contact tracing and outbreak investigation systems. Additionally, as noted elsewhere, technical and financial concerns were raised as potential barriers to sustained use of digital technologies (e.g., telehealth), particularly in primary care settings (34). As the pandemic progressed, infrastructural and equipment issues were largely overcome by organizations with the resources to do so. However, staff management persisted as a significant challenge for organizations across all settings due to an increased demand for health services and a simultaneous decrease in workforce availability. Short-term mitigation measures used to address staff shortages included redeployment or use of agency or surge workforce. While these and other strategies, such as recruitment of foreign and temporary staff and deployment of military personnel and HCWs to support civilian services, have been utilized previously in response to workforce shortages, these are not sustainable nor practical solutions (35–39). There is evidence of differential impacts of the pandemic upon healthcare occupations and settings, with areas of concern including nursing (40), primary care (41) and aged care (42). However, there is little evidence of sustainable approaches to strengthen these workforces (36, 43). As healthcare organizations transition their pandemic responses away from acute crisis management amid high rates of burnout among HCWs across the sector (2, 44, 45), effective and realistic strategies to strengthen and retain the healthcare workforce are needed (46).

HCWs identified lack of training and support in mental health and wellbeing, and infection prevention and control as key concerns. The greater physical and mental demands of work during the pandemic resulted in an increased need for camaraderie and interpersonal support. Team-building and peer-support exercises to boost staff morale were well received and could be expanded to benefit the mental health of workers in an ongoing way (47). Our findings suggest that there are problems with access to and suitability of mental health and counseling services for HCWs, which is broadly consistent with previous findings (48). In our study, HCWs cited problems accessing services due to lack of flexible scheduling arrangements and insufficient services available due to increased demand. Some HCWs felt that counseling providers possessed insufficient awareness of the challenges HCWs were experiencing to provide appropriate care. This is consistent with evidence indicating that a lack of previous experience and evidence-based services for treating front-line workers created anxiety and uncertainty for mental health professionals and led to wide variation in service provision (49). A recent systematic review indicates the need for system-wide interventions that safeguard HCWs' mental health (50). It is evident that mental health support remains a crucial aspect of the response to the COVID-19 pandemic (51), and further research is required to establish best-practice policies and protocols for establishing psychological support networks in the workplace and enable flexible and meaningful support to HCWs, both in times of crisis and beyond.

Infection control training, support and guidance was a critical element of the pandemic response. Infection control is an important factor in HCW mental health and wellbeing (52). Commonly cited barriers to good infection control practice include a lack of training or poorly communicated training (53–56). Our study found that some organizations, primarily hospitals, implemented innovative approaches to address gaps in HCW knowledge and skills and reinforce good practices such as designated PPE monitors, video-monitoring and refresher training sessions. However, consistent with previous evidence, there was a relative deficiency in the availability of adequate PPE training and respiratory fit-testing programs implemented in the primary care sector (57–59). This is of particular concern given that GPs are often the first port of call for patients experiencing symptoms of respiratory illness. It is important that lessons learnt during this pandemic are not lost in future outbreak emergencies. Universities and peak professional bodies should consider aligning medical and allied health training with PPE training guidelines to inculcate higher infection control standards (60, 61).

While similar challenges were faced across organizations, there were some differences in perceptions and impacts noted between settings and in some cases between private and public sectors. For example, while public hospitals generally had good access to PPE stockpiles and COVID-19 vaccines, other organizations did not. Additionally, the financial and business impact of government policies such as elective surgery cancellations and shifting from face-to-face to telehealth consultations disproportionately impacted private sector organizations (including primary care). This emphasizes the need for sustainable funding solutions in this sector (62). There was a general sense that greater collaboration between organizations and government was needed to ensure consistent communication and guidance across health services and to reduce inter-sector disparities.

A strength of our study was its wide sampling strategy which enabled us to capture workers with diverse views and different experiences of COVID-19 across multiple healthcare settings and organizations. Interview dialogue was strong, with lively and self-driven discussion pointing to high information power (20). Additionally, recruitment of HCWs and key personnel from the same organizations enabled a comprehensive exploration of organizational responses to the pandemic, from the dual perspective of those designing and executing such a response, rarely seen in existing literature. A limitation of our study is that interviews were conducted during a period of low COVID-19 case numbers in Victoria, along with relatively minimal restrictions, which may have led participants to provide fewer criticisms than might otherwise have been expected. Additionally, our recruitment strategy may have introduced volunteer bias, as it is possible that participants who did not indicate an interest in being interviewed differed in unpredictable ways from the selected participants. Given the evolving nature of the COVID-19 pandemic, further research is planned, including an additional round of semi-structured interviews 12-months after the interviews reported here, to examine longitudinal perceptions of organizational responses.

## Conclusions

This study provides in-depth insights into the challenges and successes of healthcare organizations' responses to the COVID-19 pandemic in Victoria, Australia, and identifies HCWs' suggestions for future responses. Future efforts to mitigate the impact of acute stressors on HCWs should include a strong focus on bidirectional communication, effective and realistic strategies to strengthen and sustain the healthcare workforce, enhancing infection control training, support and guidance, and greater investment in flexible and meaningful psychological support and wellbeing initiatives for HCWs.

## Data availability statement

The raw data supporting the conclusions of this article will be made available by the authors, without undue reservation.

## Ethics statement

This study was reviewed and approved by the Victorian Streamlined Ethical Review Process (SERP: Project Number 68086) and registered with ANZCTR (ACTRN12621000533897). The participants provided their written informed consent to participate in this study.

## COVIC-HA investigator group

The following are members of the COVIC-HA Investigator group: Peter A. Cameron, Andrew Forbes, Jane Fisher, Kelsey Grantham, Carol L. Hodgson, Peter Hunter, Jessica Kasza, Helen L. Kelsall, Maggie Kirkman, Karin Leder, Sarah L. McGuinness, Grant Russell, Philip L. Russo, Malcolm Sim, Kasha Singh, Helen Skouteris, Karen L. Smith, Rhonda L. Stuart, Helena J. Teede, James M. Trauer, Andrew Udy, Sophia Zoungas.

## Author contributions

Methodology: SM, KL, CH, JF, MK, and GR. Formal analysis: SM, OE, JF, and MK. Investigation and writing–original draft preparation: SM, KL, JJ, and OE. Resources: GR, SC, KS, CH, and HS. Data curation: SM, JJ, OE, SC, RL, HK, JF, and MK. Writing–review and editing: SM, KL, CH, SC, RL, HS, GR, MK, JF, HK, and KS. Visualization: SM and KL. Supervision: KL. Project administration: JJ and OE. Funding acquisition: SM, KL, GR, CH, HK, MK, HS, and HK. All authors have conceptualized, read, and agreed to the published version of the manuscript.

## Funding

This work was supported by the Victorian Government COVID-19 research fund (Department of Jobs, Precincts and Regions, HHSF/20/12957) and WorkSafe Victoria. The funders had no role in the study design, collection, analysis, or interpretation of the data or preparation and submission of the manuscript.

## Conflict of interest

KL (APP115500) and CH (APP1173271) were supported by National Health and Medical Research Fellowships. The remaining authors declare that the research was conducted in the absence of any commercial or financial relationships that could be construed as a potential conflict of interest.

## Publisher's note

All claims expressed in this article are solely those of the authors and do not necessarily represent those of their affiliated organizations, or those of the publisher, the editors and the reviewers. Any product that may be evaluated in this article, or claim that may be made by its manufacturer, is not guaranteed or endorsed by the publisher.
