# Supplementary material for: Organizational responses to the COVID-19 pandemic in Victoria, Australia: A qualitative study across four healthcare settings
Source: Front Public Health. 2022 Sep 29;10:965664. doi: 10.3389/fpubh.2022.965664 (PMC9557753; doi:10.3389/fpubh.2022.965664)
Supplement: Supplementary file 1 [file Data_Sheet_1.docx]

## Supplementary Table 1: Consolidated Criteria for Reporting of Qualitative Research (COREQ) Checklist

|  | **Item** | **Guide questions/description** | **Response** |
| --- | --- | --- | --- |
| ***Domain 1: Research team and reflexivity*** | | | |
| *Personal Characteristics* | *Interviewer/facilitator* | *Which author/s conducted the interview of focus group?* | Sarah McGuinness  Jane Fisher  Sharon Clifford  Riki Lane  Tess Tsindos |
|  | *Credentials* | *What were the researcher’s credentials? E.g. PhD, MD* | Sarah McGuinness – MBBS, BMedSc, DTMH, MPH&TM, FRACP, FACTM, PhD  Jane Fisher – BSc (Hons), PhD, MAPS, FCCLP, FCHP  Sharon Clifford – BSc (Hons), BA, MC, Grad.Dip.Psych  Riki Lane – BA/BSc (Hons), PhD  Tess Tsindos –MA, BA, PhD |
|  | *Occupation* | *What was their occupation at*  *the time of the study?* | Sarah McGuinness – Infectious Diseases Physician, Lecturer & Researcher  Jane Fisher – Finkel Professor of Global Health, Co-Director Division of Planetary Health  Sharon Clifford – Research Project Manager  Riki Lane – Research Fellow  Tess Tsindos – Lecturer & Researcher |
|  | *Gender* | *Was the researcher male or female?* | Male and female |
|  | *Experience and training* | *What experience or training did the researcher have?* | Sarah McGuinness is a clinician and established researcher in public health and infectious disease epidemiology, with expertise in quantitative and qualitative methods.  Jane Fisher is an academic clinical and health psychologist and an experienced public health researcher, with expertise in quantitative and qualitative methods.  Sharon Clifford is an experienced research coordinator with skills in qualitative research and long-standing connections in the Victorian primary health care network.  Riki Lane is an experienced qualitative and ethnographic researcher.  Tess Tsindos is a lecturer and teaching associate with experience in qualitative research methods.  Prior to commencement of semi-structured interviews, all interviewers attended an orientation session conducted by Maggie Kirkman (a senior qualitative researcher from the Monash University School of Public Health & Preventative Medicine). The session was recorded and circulated to the interviewing authors, for reference. |
| *Relationship with participants* | *Relationship established* | *Was a relationship established prior to study commencement?* | The study’s Project Manager (Josphin Johnson) was responsible for communications with healthcare workers (HCWs) and key personnel from the hospital, ambulance and aged care streams prior to interviews and was not involved in qualitative interviewing. Sharon Clifford from the Department of General Practice was responsible for communications with HCWs from the primary care stream and also conducted some qualitative interviews with these individuals. |
|  | *Participant knowledge of the interviewer* | *What did the participants know about the researcher? e.g. personal goals, reasons for doing the research* | Participants were provided with a written explanatory statement, which outlined the rationale and objectives of the research. Participants were informed of researchers’ positions and qualifications prior to data collection. |
|  | *Interviewer characteristics* | *What characteristics were reported about the inter viewer/facilitator? e.g. Bias, assumptions, reasons and interests in the research topic* | Participants were informed that their interview would be conducted by an experienced qualitative researcher who was not associated with the organisation that they worked for. Participants were informed that researchers conducting interviews were interested in exploring the broader effects of the COVID-19 pandemic on HCWs and exploring healthcare organisations’ responses to the pandemic from the perspective of HCWs and organisational key personnel. |
| ***Domain 2: Study design*** | | | |
| *Theoretical framework* | *Methodological orientation and theory* | *What methodological orientation was stated to underpin the study? e.g. grounded theory, discourse analysis, ethnography, phenomenology, content analysis* | This study was part of the broader Coronavirus in Victorian Aged care and Healthcare workers (COVIC-HA) cohort study, which has a longitudinal mixed methods design involving quantitative surveys and semi-structured qualitative interviews. Qualitative analysis of free-text and semi-structured interview data followed a reflexive thematic analysis approach according to the method established by Braun & Clarke (as outlined in Braun & Clarke 2019 and on the following website: https://www.psych.auckland.ac.nz/en/about/thematic-analysis.html) |
| *Participant selection* | *Sampling* | *How were participants selected? e.g. purposive, convenience, consecutive, snowball* | HCWs were sampled using purposive techniques from a pool of study participants who had completed an initial quantitative survey and provided expressions of interest in participating in the qualitative component of the study.  Due to the specific need for senior staff with knowledge and involvement in workplace responses and organisational policy implementation for key personnel interviews, healthcare organisations provided the research team with details of relevant 2-3 key personnel suitable for the interviews. Key personnel participants did not undergo sampling. |
|  | *Method of approach* | *How were participants approached? e.g. face-to-face, telephone, mail, email* | COVIC-HA participants (HCWs) were recruited via their workplaces, with an invitation to participate circulated via email by workplace CEOs or practice managers. Participants who consented to complete an initial quantitative survey were invited to express their interest in participating in a subsequent qualitative interview. Following purposive sampling, selected participants were contacted by project manager Josphin Johnson or project assistant Sharon Clifford via phone/email and provided with a detailed explanatory statement and a list of potential interview times. Once a time was confirmed, participants were sent a calendar invite and Zoom video conference link.  Key personnel were also recruited via their workplaces. Investigators or CEOs from each participating site were requested to nominate 2-3 senior personnel with key knowledge about their respective organisation’s preparedness and response to the COVID-19 pandemic. Nominated key personnel were contacted by project manager Josphin Johnson following the same procedure as HCW participants. |
|  | *Sample size* | *How many participants were in the study?* | 28 HCWs and 21 key personnel participated in qualitative interviews. 365 participants provided free-text responses from the COVIC-HA baseline survey. |
|  | *Non-participation* | *How many people refused to participate or dropped out? Reasons?* | 1 HCW who was approached did not proceed with an interview due to other commitments (dropped out). All other HCWs who were approached for interviews consented to participate.  1 key personnel participant who was scheduled for an interview could not proceed due to unanticipated conflicting time commitments. All other key personnel who were approached for interviews consented to participate. |
| *Setting* | *Setting of data collection* | *Where was the data collected? e.g. home, clinic, workplace* | Interview data was collected via the video-conferencing platform Zoom; individual sessions were recorded with participants’ consent. Participants could choose to join the video conference from their home or workplace as suited them.  Free-text responses to the question “What resources or supports would you like to see your organisation put in place to support you during the COVID-19 pandemic or any future crisis events?” were obtained from the COVIC-HA baseline survey, conducted online via REDCap. |
|  | *Presence of non-participants* | *Was anyone else present besides the participants and researchers?* | No |
|  | *Description of sample* | *What are the important characteristics of the sample? e.g. demographic data, date* | For the purposes of the qualitative component of the research, demographic data obtained and reported was age, gender, work stream and work experience, study site location and exposure to COVID-19. |
| *Data collection* | *Focus group guide and interview guide* | *Were questions, prompts, guides provided by the authors? Was it pilot tested?* | Two interview topic guides were developed separately for HCWs and key personnel and each were piloted within the study’s investigator group prior to interview commencement. The topic guides included a series of open-ended questions and prompts but allowed interviewers to alter the sequence of questions or the way in which they were phrased. Participants were not provided with the topic guide prior to interviews. |
|  | *Repeat interviews* | *Were repeat interviews carried out? If yes, how many?* | No |
|  | *Audio/visual recording* | *Did the research use audio or visual recording to collect the data?* | Interviews were conducted via video-conference, with audio recording enabled. A professional transcription service was engaged to transcribe audio recording into word documents. Following transcription, a member of the research team assessed the transcripts against the original recording for accuracy. |
|  | *Field notes* | *Were field notes made during and/or after the interview or focus group?* | Researchers made field notes during interviews. |
|  | *Duration* | *What was the duration of the inter views or focus group?* | Interview duration was dependent upon how much the participant wanted to say. Average interview duration across the 28 HCW and 21 key personnel interviews was 36 minutes with a range of 19-59mins among HCW interviews and 18-60mins among key personnel interviews. |
|  | *Data saturation* | *Was data saturation discussed?* | The concept of data saturation is not consistent with the epistemological approach of our research. Our approach was guided by the concept of information power, which aims to provide robust information directly related to the research question. Therefore, we chose not to attempt to ‘capture’ data saturation. Instead, we aimed to purposively recruit diverse participants who presented a wide range of views that answered the research question. Participants were from a range of different age groups, genders, professions and work streams; participants with different levels of COVID-19 exposure (e.g. infected, furloughed) and participants with positive and negative views of organisational responses were represented. |
|  | *Transcripts returned* | *Were transcripts returned to participants for comment and/or correction?* | No |
| ***Domain 3: Analysis and findings*** | | | |
| *Data analysis* | *Number of data coders* | *How many data coders coded the data?* | Three |
|  | *Description of the coding tree* | *Did authors provide a description of the coding tree?* | Yes. Figure 2 provides an illustration of the source of themes and sub-themes in our research. |
|  | *Derivation of themes* | *Were themes identified in advance or derived from the data?* | Themes were derived inductively from the data following an established methodology stipulated by Braun & Clarke. |
|  | *Software* | *What software, if applicable, was used to manage the data?* | NVivo version 11 |
|  | *Participant checking* | *Did participants provide feedback on the findings?* | No |
| *Reporting* | *Quotations presented* | *Were participant quotations presented to illustrate the themes/findings? Was each quotation identified? e.g. participant number* | Participant quotations from each data source were included in findings. The source of data, profession and study setting of the quoted individual is indicated for each quotation. A unique and de-identified study code was assigned to each participant to identify the source of quotations (e.g. HCWI08 = Healthcare worker participant, interview 8) |
|  | *Data and findings consistent* | *Was there consistency between the data presented and the findings?* | Yes |
|  | *Clarity of major themes* | *Were major themes clearly presented in the findings?* | Yes |
|  | *Clarity of minor themes* | *Is there a description of diverse cases or discussion of minor themes?* | Yes. Themes and their inherent subthemes are reported in this study from a diverse set of participants. |

## Supplementary Figure 1: Timeline of COVIC-HA Study within the context of the COVID-19 pandemic in Victoria, Australia


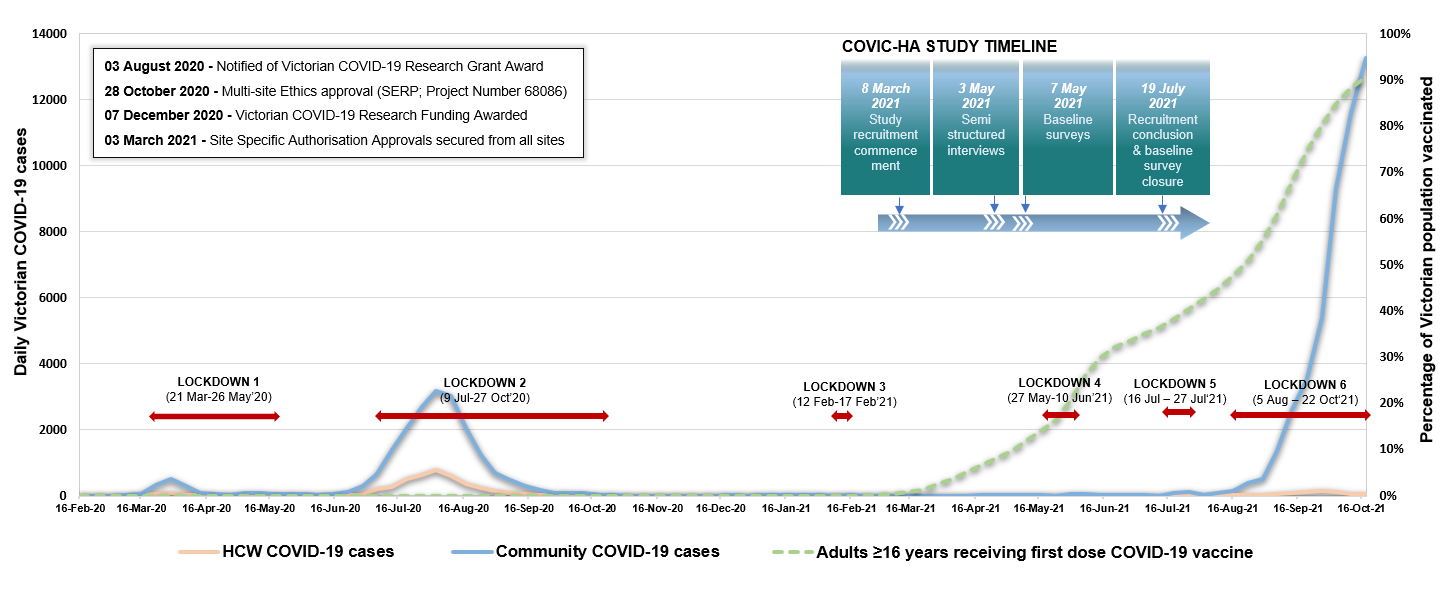
 COVID-19 case numbers sourced from Victorian COVID-19 data available at <https://www.coronavirus.vic.gov.au/victorian-coronavirus-covid-19-data> (accessed on 18 November 2021); Victorian healthcare worker (clinical and non-clinical) COVID-19 data available at <https://www.coronavirus.vic.gov.au/healthcare-worker-covid-19-data> (accessed on 18 November 2021). COVID-19 vaccine data for Victoria were sourced from <https://www.coronavirus.vic.gov.au/weekly-covid-19-vaccine-data#covid-19-vaccine-rates-for-second-dose-by-postcode> (accessed on 18 November 2021).

**Supplementary File 1:** Healthcare worker (HCW) semi-structured interview guide

**Opening (preamble)**

- Introduce yourself and thank the participant
- Do I have your permission to begin recording?
- Explain purpose: to learn about your experiences during COVID-19 and your perceptions of your organisation’s response
- Any questions after reading the explanatory statement?
- Do you consent to participating in the interview?

**Professional role and impact of COVID-19 on work role/identity**

Can you tell me a little about your professional role?

How has COVID-19 impacted you as a healthcare worker?

- Potential prompts (if needed): Work role? Sense of identity? Mental health? Confidence?

**Challenges and managing them**

Can you tell me about any challenges you’ve faced during the COVID-19 pandemic?

- At work? At home? Financial? Feelings of isolation? Separation from family?
- What helped? What made it hard?
- What did your organisation do to support you? What else could they have done?

**Exposure to COVID-19; experience of isolation, quarantine**

Can you talk about any contact you had with COVID-19 cases at work, and how you felt about this?

- Caring for cases? Personal safety? Fear of exposure
- If isolated/quarantined – experiences with/feelings about this?
- What did your organisation do to support you? What else could it have done?

**COVID-19 Vaccines**

Can you tell me about your experiences with or feelings about COVID-19 vaccination?

- Which vaccine received/offered? How easy/hard was it to get? How did it make you feel?
- What has come up in discussions with others (friends, family, colleagues) about vaccines?
- Have discussions with others / news media influenced your thoughts/choices?

**Organisational response**

Can you tell me your thoughts on your organisation’s response to COVID-19?

- What resources or supports have helped? How?
- What was unhelpful / made things harder?
- What else could be done to protect or support you?
- Were there specific activities that helped to boost staff morale?

**Opportunities**

Can you tell me about any positive experiences you’ve had during the COVID-19 pandemic?

- At work? At home? New opportunities? New hobbies?

***Is there anything else you’d like to say about your experiences of COVID-19?***

**Closing**

- Thank participant for their contribution
- If you think of something you wish you’d said, please email [study address] and we will add this information to your transcript

**Supplementary File 2:** Key informant semi-structured interview guide

**Opening (preamble)**

- Introduce yourself and thank the participant for making time available to talk to us
- Explain purpose of interview: to learn about the way your organisation prepared for and has responded to the COVID-19 pandemic.
- Do you have any questions after reading the explanatory statement?
- Do I have your permission to record this conversation?

**Professional role**

Can you tell me a little about your role in your organisation?

**Impacts of the pandemic**

Can you describe some of the key impacts/effects that COVID-19 has had on your organisation?

- Challenges? Opportunities? Changes in care provision / structure?
- How have changes been communicated to staff?

**Worker safety and support**

What have been some of the key changes your organisation has made to manage the risk of COVID-19 transmission in the workplace?

- Which have been most effective / important / well-received?

What things has your organisation done to support workers +/- boost morale during the pandemic?

**Personal protective equipment (PPE) for workers**

- What changes has your organisation made to PPE training and auditing as a result of COVID-19 (e.g. format/mode, training providers, frequency, fit testing use of ‘spotters’)
- Are there any current issues of concern regarding PPE for workers that your organisation is facing, for example in terms of availability, training, policy development, compliance or monitoring of compliance?

**COVID-19 vaccination rollout**

Can you tell me about your organisation’s experience with the COVID-19 vaccine rollout so far?

- Barriers/enablers to uptake/acceptance? Logistical challenges?

**Preparation and planning**

What outbreak preparations and plans did your organisation have in place prior to the COVID-19 pandemic?

- What aspects of these preparations/plans were most useful in responding to COVID-19?
- What could be improved in future plans and procedures?

**Lessons**

What lessons has your organisation learned from COVID-19 so far?

***Is there anything else you’d like to say about your organisation’s experiences of COVID-19?***

**Closing**

- Thank you for your valuable contribution
- If you think of something you wish you’d said please email [study address] and we will add this information to your transcript
